# Paging through history: parchment as a reservoir of ancient DNA for next generation sequencing

**DOI:** 10.1098/rstb.2013.0379

**Published:** 2015-01-19

**Authors:** M. D. Teasdale, N. L. van Doorn, S. Fiddyment, C. C. Webb, T. O'Connor, M. Hofreiter, M. J. Collins, D. G. Bradley

**Affiliations:** 1 Smurfit Institute of Genetics, University of Dublin, Trinity College, Dublin 2, Ireland; 2 BioArCh, University of York, York YO10 5DD, UK; 3 Borthwick Institute for Archives, University of York, York YO10 5DD, UK; 4 Institute for Biochemistry and Biology, Faculty of Natural Sciences, University of Potsdam, Karl-Liebknecht-Str. 24–25, Potsdam 14476, Germany

**Keywords:** parchment, next generation sequencing, ancient DNA, ZooMS, sheep

## Abstract

Parchment represents an invaluable cultural reservoir. Retrieving an additional layer of information from these abundant, dated livestock-skins via the use of ancient DNA (aDNA) sequencing has been mooted by a number of researchers. However, prior PCR-based work has indicated that this may be challenged by cross-individual and cross-species contamination, perhaps from the bulk parchment preparation process. Here we apply next generation sequencing to two parchments of seventeenth and eighteenth century northern English provenance. Following alignment to the published sheep, goat, cow and human genomes, it is clear that the only genome displaying substantial unique homology is sheep and this species identification is confirmed by collagen peptide mass spectrometry. Only 4% of sequence reads align preferentially to a different species indicating low contamination across species. Moreover, mitochondrial DNA sequences suggest an upper bound of contamination at 5%. Over 45% of reads aligned to the sheep genome, and even this limited sequencing exercise yield 9 and 7% of each sampled sheep genome post filtering, allowing the mapping of genetic affinity to modern British sheep breeds. We conclude that parchment represents an excellent substrate for genomic analyses of historical livestock.

## 1. Introduction

Before the mass production of paper, parchment was the major medium for codices and until the widespread adoption of typewriters, they were a clerk's preferred medium for many formal legal documents and records [1]. There are several aspects of parchments that mark them as compelling substrates for DNA extraction and analysis. Firstly, parchments are made from the skins of domestic animals, particularly cattle, sheep and goats, which are dehaired, stretched, dried, scraped and pounced [1,2]. This manufacturing process results in robust artefacts, which can survive intact for many centuries [1,2]. Secondly, parchments/parchment manuscripts are not only abundant and widespread, but because of their enduring legal and evidential value they have typically been carefully managed throughout their lives and, in the twentieth century, curated and protected from both high temperatures and fluctuating humidity. Indeed, the number of skins is truly staggering, even if, as is likely, a high percentage of documents have been destroyed. In the UK alone, assuming the number of sheep slaughtered annually remained constant at 15 million from 1150 to 1850 [3], then if only 1% of all the skins became parchment and only 4% survived, this would equate to 4.2 million animals' skins [3,4]. It is, however, difficult to estimate the total number of parchment documents, but the total number surviving in the UK must be well in excess of one million items. Thirdly, unlike bone remains, of which only a fractional percentage survives and much less have been excavated, all the skins are above ground, archived and in the case of legal documents, directly dated to specific calendar years and, usually, precise days, which is a level of resolution not readily achievable with any other historic DNA source. Even documents that do not carry a direct date can be dated palaeographically to a resolution better than radiocarbon dating (without the expense of this process) [5]. Finally, there is enormous interest in the genetics of the main parchment species, cattle, sheep and goat, each with vibrant research communities investigating both geographical and temporal genetic variation [6–8].

Although understudied, parchment has been the subject of prior ancient DNA (aDNA) research. For example, the Dead Sea scrolls have yielded mitochondrial DNA PCR fragments, which have been identified as ibex and goat [9,10]. Similarly, Poulakakis *et al.* [11] identified three thirteenth to sixteenth century Greek parchments as of goat origin. Promisingly, Burger *et al.* [12,13] also recovered autosomal DNA amplicons in addition to mtDNA from parchments, suggesting the possibility of high-resolution genetic inference. However, one recent study has been less encouraging. Campana *et al.* [2] investigated eighteenth to nineteenth century British parchments and found that a majority of these gave heterogeneous mtDNA amplification products with signatures from multiple individuals and species, in addition to a high proportion of chimeric PCR artefacts. This result was attributed to cross-contamination in the industrial parchment production process, during which multiple animal skins may have been washed, cured and depilated together [2].

However, PCR-based aDNA research has well-documented deficiencies, particularly with regard to controlling and estimating contamination [14,15]. A central issue is that PCR favours longer, less damaged templates and thus has a bias for contaminant over endogenous DNA, making a representative sampling of target molecules impossible. By contrast, next generation sequencing (NGS) of aDNA works well with shorter fragments, generates many orders of magnitude more data, shows greater sensitivity, including the analysis of autosomal DNA, and is less prone to the chimeric artefactual sequences that can emerge from PCR [16]. Already, NGS of ancient nuclear DNA has provided insights into the evolutionary history of both extant and extinct species [17–21], but the routine analysis of ancient nuclear genomes is limited by the availability of well-preserved historic and archaeological samples [22,23]. The main limitation for the analysis of bone specimens lies in the fact that most palaeontological and archaeological samples are found to contain high levels of bacterial and low levels of endogenous DNA (approx. 0.1–5%) [18,19,21,24], although there are some notable high profile exceptions [20].

Here we present a molecular archaeological analysis of parchment using two historical samples from the Borthwick Institute for Archives at the University of York dated palaeographically to the seventeenth (PA1) and eighteenth (PA2) century, respectively. Both of these samples are identified as sheep and give high proportions of endogenous DNA with very low contamination from other species or co-specific individuals, suggesting parchment as an excellent source of historic DNA. Following a conventional agricultural history narrative, PA1 predates the livestock ‘improvements' driven by Bakewell and Ellman among others, while PA2 falls into the period when the new breeds of sheep were being developed and spread. The variation uncovered shows the potential of genetics in localizing the geographical origins of artefacts.

## 2. Material and methods

### (a) Zooarchaeology by mass spectrometry

Parchment samples of circa 5 × 5 mm from PA1 (seventeenth century) and PA2 (eighteenth century), were obtained from the Borthwick Institute for Archives parchment discards, and incubated twice for 1 h in 50 mM ammonium bicarbonate buffer (pH 8.0) at 65°C following the method of van Doorn *et al.* [25]. The first extract was discarded and the second extract was trypsinated overnight at 37°C. The tryptic digest was transferred over C18 resin to desalt and concentrate peptides by washing with 0.1% trifluoroacetic acid (TFA). Peptides were eluted in a final volume of 10 μl of 50% acetonitrile (ACN)/0.1% TFA (v/v). A measure of 1 μl of elute was mixed on a ground steel plate with 1 μl α-cyano-4-hydroxycinnamic acid matrix solution (1% in 50% ACN/0.1% TFA (v/v/v)) and air-dried. Samples were analysed using a calibrated Ultraflex III MALDI-TOF instrument in reflector mode. Peptides were identified manually according to Buckley *et al.* [26,27]. Campana *et al*. [28] have confirmed the ability of collagen to discriminate sheep and goat [27] using DNA sequencing.

### (b) Ancient DNA extraction

All DNA extractions were performed in a dedicated aDNA laboratory at Trinity College, Dublin, on the same parchment samples used for zooarchaeology by mass spectrometry (ZooMS). Given the pilot nature of this analysis, the maximum amount of starting material available was used for each extraction, with DNA retrieved from 2 × 2 cm^2^ (approx. 0.05 g) pieces cut from both parchments, prepared using procedures previously described by our group [29,30].

### (c) Illumina sequencing library preparation

Illumina single read sequencing libraries were produced for each of the two parchment samples via PCR amplification of end-repaired-adapter-ligated DNA templates following [29]. Samples were indexed following the Craig *et al.* [31] method of barcoding. Two PCR amplifications (20 μl) were performed for each enrichment step comprising 3 μl of end-repaired-adapter-ligated parchment DNA, 10 μl Phusion high-fidelity PCR master mix with HF buffer 6.2 μl ddH_2_O and 0.4 μl each of both the forward and reverse primers. Amplification reactions per library consisted of an initial denaturation step of 98°C for 30 s, then 12 cycles of 98°C for 10 s, 65°C for 30 s and 72°C for 30 s, followed by a final extension step of 72°C for 5 min. The final PCR products from each sample (two each) were then pooled and visualized on an Agilent 2100 Bioanalyzer using a DNA 1000 chip. Both samples were then combined in equimolar ratios and sequenced together on a single (SE 49 bp) lane of an Illumina HiSeq2000 at BGI.

### (d) Initial parchment sequencing quality control

Initial quality control of sequencing reads were performed using FastQC (http://www.bioinformatics.babraham.ac.uk/projects/fastqc/). Adapter sequences were trimmed from the 3′ end of the reads using Cutadapt [32]. Cutadapts default settings were modified to require only a 1 bp overlap between the 3′ sequence of a read and an adapter sequence for it to be trimmed. This highly conservative approach will lead to a high proportion of reads being trimmed due to spurious matches. However, this approach was selected to try to guard against subsequent adapter sequence poisoning of downstream analyses, which can lead to poor alignment of reads and misidentification of sequence polymorphisms.

### (e) FastQ Screen

FastQ Screen (http://www.bioinformatics.babraham.ac.uk/projects/fastq_screen) is a Perl wrapper script, which allows for the same sequencing library to be easily aligned to multiple reference genomes using Bowtie [33]. The percentage of raw parchment reads that aligned uniquely to a single genome, to multiple places in the same genome, uniquely in multiple genomes and to multiple places in multiple genomes can then be assessed. FastQ Screen was used in this analysis to align the trimmed reads (30 bp minimum read length) to four genomes, sheep (oviAri3), cow (bosTau7), human (hg19) and goat (chir1). FastQ Screen alignment settings were modified to use Bowtie's ‘end-to-end’ algorithm and to allow the number of mismatches between the read and the reference genome to vary between 0 and 3.

### (f) BWA sequence alignment

The raw trimmed reads (30 bp minimum read length) were also aligned to the sheep reference genome (oviAri3) minus the mitochondrial genome [21] using BWA [34]. Standard alignment settings for the use of BWA with aDNA were used [35]. Aligned reads were then further filtered for a minimum mapping quality of 30 and redundant clonal PCR amplified reads removed using the SAMtools rmdup command [36]. Uniquely aligned reads were then identified by the XT, X1 tags, produced from the BWA alignment.

### (g) Alignment to human genome and variant calling

All sequencing reads were further aligned to the human genome (hg19) using BWA and SAMtools with identical parameters to the sheep (oviAri3) alignments above. Any reads that aligned to the human genome were then removed from subsequent analysis irrespective of mapping quality. SNPs were called using established protocols for aDNA NGS data [18,24]. Briefly, SNPs were called for all positions in which the shotgun sequencing of the parchment overlapped with the positions of SNPs in the ovine HapMap dataset (oviAri3 alignment), requiring a minimum base quality of 15 and mapping quality of 30. If multiple reads overlapped a SNP position, one read was then taken at random and used for base calling; C/T and G/A SNPs were also removed.

### (h) SNP merging and allele sharing

The SNPs called from PA1 and PA2 were then doubled to create pseudo-diploid data and merged with data from the sheep HapMap (ovine 50K panel, electronic supplementary material, table S2) [6] using PLINK [37]. PA1 and PA2 SNPs were flipped to match the orientation of the HapMap with A/T and G/C SNPs removed from the analysis. Allele sharing distances were then calculated using a custom Python script and visualized using the ArcMap software in the ArcGis suite (Environmental Systems Research Institute).

### (i) Whole mitochondrial genomes

Full mitochondrial genomes were produced from alignments of the parchment reads to a modern mitochondrial reference genome (HM236176) with BWA. Redundant reads were then removed using SAMtools. Modern sheep mitochondrial reference genomes were downloaded from GenBank (*n* = 23) to allow the placement of the parchment samples within this dataset. Multiple sequence alignments of both the modern and parchment mtDNA genomes were completed using the MUSCLE alignment algorithm [38] implemented in SeaView [39]. Neighbour joining trees (1000 bootstrap replicates) were then produced from the alignment data in SeaView using the default Jukes and Cantor model and ignoring gapped sites.

## 3. Results

### (a) Species identification

**Figure 1.**
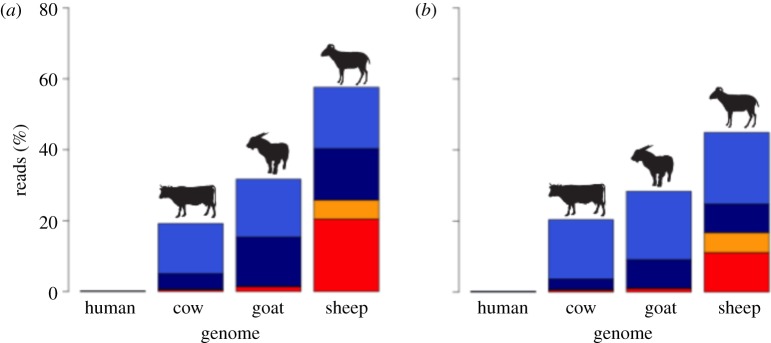
Histograms illustrating the relative frequency of raw sequence read alignments to the human, cow, sheep and goat genomes with a tolerance of zero mismatches. (*a*) PA1 and (*b*) PA2, red, one hit one genome, orange, multiple hits one genome, dark blue, one hit multiple genomes, blue, multiple hits multiple genomes. Notably, only the sheep genome shows a significant body of aligning reads that do not also align to other species. Cross alignment of other reads is expected owing to the high homology, especially of repeated elements, among ruminant genomes. (Online version in colour.)

Identification of the source species of each parchment was completed using a combined proteomic/genomic approach. The results of both these analyses identified sheepskin as the likely origin (figure 1; electronic supplementary material, figures S1 and S2). After re-alignment to the sheep genome and filtering for mapping quality and contamination (reads that aligned the human genome) a final set of 6 047 847 (35.5%) reads were retained for PA1 and 5 256 723 (16.7%) for PA2, which equates to a retrieval of 9.4 and 7.9% of the sheep genome in PA1 and PA2, respectively. Both parchments were also identified as being produced from ewes via the analysis of the ratio of X chromosome to autosome reads [21].

### (b) Mitochondrial DNA analysis

In total, 25 314 reads aligned to the ovine mitochondrial genome after duplicate removal (table 1; PA1 = 11 271, PA2 = 14 043). This allowed for the production of whole mitochondrial genomes from both samples with an average read depth of 28X and 33X, respectively. These genomes were then compared to a modern reference dataset (*n* = 23; electronic supplementary material, table S1) including 17 domestic sheep and six other ovine samples. Both parchments were found to locate within the domestic sheep mitochondrial haplogroup B (electronic supplementary material, figure S3), which is predominant in both the modern and ancient sheep populations of Europe [40,41].

**Table 1.** Summary of ancient sequence data from both parchment samples.

sample	raw reads	aligned reads raw (%)	aligned reads high quality^a^ (%)	aligned reads mtDNA	ovine 50K panel SNPs called
PA1 (seventeenth century)	17 006 629	11 292 116 (66.4)	6 047 847 (35.5)	11 271	3168
PA2 (eighteenth century)	31 493 502	14 403 079 (45.7)	5 256 723 (16.7)	14 043	2291

^a^High quality reads consists of a mapping quality greater than or equal to 30, no reads which also align to the human genome (hg19) and unique as described by the XT and X1 tags of the BWA mapping algorithm.

The high level of mitochondrial genome coverage achieved in the parchment sequencing allows for a rough estimation of the contamination rate in these samples to be calculated (both historic and modern). To do this, haplotype informative polymorphic positions in both samples, outside the difficult to align 75–76 bp repeat motif located within the control region of the ovine mtDNA [42,43], that may be due to contamination, sequencing error, heteroplasmy or DNA damage were analysed [24]. At these positions, we found the sample consensus base in 96% of sequences (282/291) for PA1 and 95% (381/398) of sequences for PA2, giving maximum estimated contamination rates of 4% for PA1 and 5% for PA2. These figures are slightly less than in the study by Sánchez-Quinto *et al.* [24] of a complete ancient human mitochondrial genome which, using similar methods, estimated exogenous contamination at 8%.

### (c) SNP analysis

**Figure 2.**
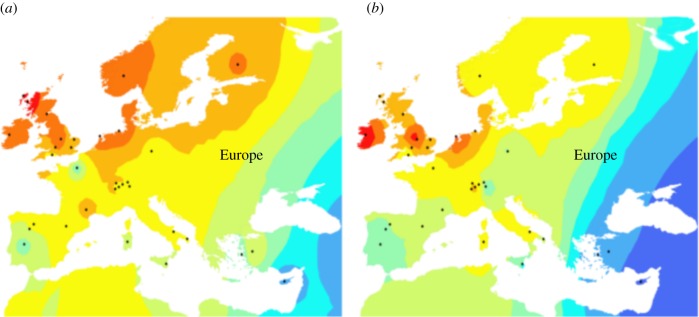
Synthetic maps illustrating average allele sharing between the two parchment partial genome sequences and reference genotypes from selected modern sheep breeds. Higher sharing is denoted by warmer colours. A localization of genetic affinity for both to western Europe is clear, (*a*) PA1 showing more sharing with northern Britain and (*b*) PA2 with southern Britain and Ireland. Approximate geographical origin of breeds from Kijas *et al.* [6]. (Online version in colour.)

Both parchment samples were genotyped following established protocols for aDNA data [18,24]. A total of 3168 SNPs from the ovine 50K panel were called for PA1 and 2291 for PA2 after filtering and merging with the modern genotype data. Average allele-sharing scores between each parchment genotype and extant geographically sampled populations were calculated and are summarized graphically in the interpolated contour maps in figure 2. Despite the limited recovery of SNP genotypes, a localization towards the British Isles is seen.

## 4. Discussion

Parchment is ubiquitous in the historical record and is an attractive source for aDNA analysis to elucidate the history of domestic species and address questions intrinsic to the material origins of documents. However, published reports of first generation parchment aDNA investigations indicate conflicting results, reporting both unique PCR amplification products from a single species [11,13,44] and, more recently, multiple sequences from multiple species [2]. The latter finding seemed to imply cross-contamination between skins during the preparation process, where multiple animals would have been co-treated, a result potentially fatal for successful analysis.

In contrast to these results, our samples showed no signs of manufacture contamination in either proteomic or aDNA approaches. ZooMS identified sheepskin as the likely origin for both parchments and failed to retrieve any taxon-specific collagen masses for goat, pig or cow. FastQ Screen analysis of the raw DNA sequencing data identified the sheep genome as the most likely source for the majority of the sequences. This analysis also identified relatively few molecules that aligned uniquely to either the bovine, goat or human genome at zero mismatches; 1.0, 2.4 and 0.02% of all reads, respectively (figure 1; electronic supplementary material, table S3). These non-source species alignment percentages are likely caused by homology between the ruminant genomes as well as damage in the aDNA molecules producing a better alignment to non-source species. Moreover, these percentages are likely to be inflated by the differences in the completeness of genome builds between the species [8], with the ovine genome being one of the least complete.

The high coverage mitochondrial genomes also allowed for the estimation of a within species contamination rate for both samples, which were found to be lower than that previously reported for human aDNA samples at 8% (4% for PA1 and 5% for PA2) [24]. Undetermined mismatch errors due to sequence misreads and DNA damage may also have contributed to these proportions, suggesting that the real contamination rate is even lower. In summary, the analysis of our shotgun data suggests that they were, at most, affected by low levels of contamination either from different individuals of the same species (sheep) or some of the most commonly contaminating species, human, goat and cow. It should also be noted that both the above analyses (FastQ Screen and mtDNA) were completed on unfiltered data and are therefore likely to be unbiased by the high level of filtering conducted for the SNP analysis. Thus, our results are in agreement with some previous studies suggesting parchment as a valuable source for historic DNA sequences, but contrast with the most recent work [2] that suggested high levels of contamination and artefacts in PCR-based DNA analyses from parchment. It should be noted that many contaminant sequences detected by Campana *et al.* [2] were unusual, and the authors note the possibility of artefactual origin such as jumping PCR.

In order to provide as pure a sheep dataset as possible, stringent filters were applied in a re-alignment of all reads to the sheep genome to remove putative human contaminants. For both parchment samples more than 45% of reads aligned to the ovine genome once clonally amplified products were removed (table 1). These numbers decrease to 35.5% (PA1) and 16.7% (PA2) when filters for mapping quality (minimum mapping quality = 30), possible human contamination and uniquely aligned reads are applied. These values, however, still equate to a retrieval of 9.4 and 7.9% of the sheep genome in PA1 and PA2, respectively. These mapping percentages are still very high in comparison to those obtained for bone extracts, which are typically in the range 0.1–5%. This large percentage of endogenous DNA is likely facilitated by the parchments' lower age, the nature of the source material (skin instead of bone) and the preferential conditions in which they were stored. This analysis suggests that parchments are an excellent source of historic DNA and are superior to bones and teeth of a similar age in the amount of endogenous DNA retrieved and resolution of the dating available, reliable dating being critical in the analysis of the effects of agricultural selection.

If other parchments show similar levels of endogenous DNA content, DNA sequencing of historic domesticated animal genomes over a range of time periods could be accomplished, providing insights into the breeding history of domesticated animals; for example, into sheep breeding before, during and after the agricultural improvements of the eighteenth century that led to the emergence of regional breeds of sheep in Britain.

Indeed, it is intriguing to note that the two items, both of them records held in the Borthwick Institute for Archives in York, seem to have different heat map distributions, which may represent different breeding strategies employed between these two time points [45,46]. The visualization of the genetic affinities of our two samples offers an illustration of the potential of co-analysis of dense modern SNP genotypes with next generation aDNA data. PA1 shows a strong affinity with northern Britain, specifically the region in which black-faced breeds such as Swaledale, Rough Fell and Scottish Blackface have a deep history [45]. The affinity with modern Texel sheep is intriguing in view of a rare retroviral insertion event seen in Texels and in two historic northern English breeds [47]. PA2 shows closer affinity with the Midlands and southern Britain, the region in which the livestock improvements of the later eighteenth century were most active. Although this is somewhat speculative, the two specimens may derive from an unimproved northern hill-sheep (PA1), as might be expected in Yorkshire in the seventeenth century, and a sheep derived from the ‘improved’ flocks that were spreading through England in the eighteenth century, predominantly from estates in the Midlands (PA2). Although selected for a proof-of-principle investigation, these two documents may have given us a snapshot of livestock improvement in process. This is a controversial period in agricultural history, specifically around the issue of whether the livestock improvers developed new strains *de novo*, or built on changes that were already in progress [48]. As a productive and inherently datable biomolecular source, parchments will enable a more nuanced analysis of livestock regionalisation, one that complements the documentary record and is more chronologically precise than the archaeological record alone.

## 5. Conclusion

The findings of this first NGS study of parchment have shown that parchment is a highly suitable substrate for large-scale aDNA analyses. We were able to retrieve 9 and 7% of the ovine genome from PA1 and PA2, respectively, at high quality (mapping quality ≥ 30) using just half a lane each of an Illumina HiSeq 2000. This result suggests that the production of whole historic domesticate genomes or targeted exon sequencing from parchment is a realistic possibility. We were able to provide a unique species assignment for both pieces using both proteomic and aDNA methods, and estimate an external contamination rate comparable to, and probably lower than from, other aDNA sources. Whole mitochondrial genomes produced from both samples allowed their placement within the most populous sheep haplogroup, and SNP data allowed an estimation of the modern day sheep breeds that most closely resemble the historic samples to be identified. Further sequencing of parchment samples from a variety of time periods and locations should allow for genetic maps from a variety of domestic species to be built, providing important insights into the past 1000 years of animal breeding history.

## Supplementary Material

Supplementary figures and tables

## Data accessibility

Raw sequence data can be found at the European Nucleotide Archive, study accession PRJEB5933. http://www.ebi.ac.uk/ena/data/view/PRJEB5933.
