# Supplementary material for: Paging through history: parchment as a reservoir of ancient DNA for next generation sequencing
Source: Philos Trans R Soc Lond B Biol Sci. 2015 Jan 19;370(1660):20130379. doi: 10.1098/rstb.2013.0379 (PMC4275887; doi:10.1098/rstb.2013.0379)

Teasdale, MD *et al*. 2015 Paging through history: parchment as a reservoir of ancient DNA for next generation sequencing. *Phil. Trans. R. Soc. B.* **370** doi: 10.1098/rstb.2013.0379

**Supplementary Table 1: Sheep sequences used for tree drawing (Supplemental figure 3)**

| Accession number | Haplogroup | Breed stated in GenBank record |
|---|---|---|
| HM236175 | A | Romney |
| HM236174 | A | Merino |
| NC_001941 | B | Merinolandschaf |
| HM236177 | B | Karakas |
| HM236176 | B | Karakas |
| EF490456 | B | Finn Dorset |
| EF490454 | B | Finn Dorset |
| EF490452 | B | Finn Dorset |
| EF490455 | B | Finn Dorset |
| EF490453 | B | Finn Dorset |
| EF490451 | B | Finn Dorset |
| HM236179 | C | Morkaraman |
| HM236178 | C | Karakas |
| HM236181 | D | Morkaraman |
| HM236180 | D | Morkaraman |
| HM236183 | E | Tuj |
| HM236182 | E | Awassi |
| HM236186 | *O.vignei* | *N/A* |
| HM236185 | *O. musimon* | *N/A* |
| HM236184 | *O. musimon* | *N/A* |
| HM236187 | *O.vignei* | *N/A* |
| HM236188 | *O. ammon* | *N/A* |
| HM236189 | *O.vignei* | *N/A* |

**Supplementary Table 2: Sheep breeds used in genetic distance analysis**

| ID | 3 letter abbreviation Ovine HapMap | Regional origin as defined by Kijas *et al* (2012) |
|---|---|---|
| African Dorper | ADP | Africa |
| African White Dorper | AWD | Africa |
| Afshari | AFS | SW Asia |
| Altamurana | ALT | SW Europe |
| Australian Industry Merino | MER | SW Europe |
| Australian Suffolk | ASU | Northern Europe |
| Bangladeshi BGE | BGE | Asia |
| Bangladeshi Garole | BGA | Asia |
| Border Leicester | BRL | Northern Europe |
| Boreray | BOR | Northern Europe |
| Bundner Oberlander Sheep | BOS | Central Europe |
| Castellana | CAS | SW Europe |
| Changthangi | CHA | Asia |
| Chios | CHI | SW Europe |
| Churra | CHU | SW Europe |
| Comisana | COM | SW Europe |
| Cyprus Fat Tail | CFT | SW Asia |
| Deccani | IDC | Asia |
| Dorset Horn | DSH | Northern Europe |
| East Friesian White | EFW | Central Europe |
| Engadine Red Sheep | ERS | Central Europe |
| Ethiopian Menz | EMZ | Africa |
| Finn sheep | FIN | Northern Europe |
| Galway | GAL | Northern Europe |
| Garut | GUR | Asia |
| German Texel | GTX | Northern Europe |
| Indian Garole | GAR | Asia |
| Karakas | KRS | SW Asia |
| Leccese | LEC | SW Europe |
| Merino Landschaf | MLA | SW Europe |
| Milk Lacaune | LAC | SW Europe |
| Moghani | MOG | SW Asia |
| Namaqua Afrikaner | NQA | Africa |
| New Zealand Romney | ROM | Northern Europe |
| Norduz | NDZ | SW Asia |
| Old Norwegian Spaelsau | NSP | Northern Europe |
| Qezel | QEZ | SW Asia |

| | | |
|---|---|---|
| Rambouillet | RMB | SW Europe |
| Rasa Aragonesa | RAA | SW Europe |
| Red Maasai | RMA | Africa |
| Sakiz | SKZ | SW Asia |
| Sardinian Ancestral Black | SAB | SW Europe |
| Scottish Blackface | SBF | Northern Europe |
| Soay | SOA | Northern Europe |
| Spael-coloured | NSP | Northern Europe |
| Spael-white | NSP | Northern Europe |
| Sumatra | SUM | Asia |
| Swiss Black-Brown Mountain Sheep | SBS | Central Europe |
| Swiss Mirror Sheep | SMS | Central Europe |
| Swiss White Alpine Sheep | SWA | Central Europe |
| Tibetan | TIB | Asia |
| Valais Blacknose Sheep | VBS | Central Europe |
| Valais Red Sheep | VRS | Central Europe |
| Wiltshire | WIL | Northern Europe |

From Kijas *et al*. (2012). South-West Asia (SW Asia) refers to present day Turkey and Iran, also sometimes referred to as the Middle East.

**Supplementary Table 3: Unique alignment percentages to possible contaminant genomes at 0 mismatches from FastQ Screen.**

| ***Genome*** | ***Par1*** | ***Par2*** |
|---|---|---|
| | | |
| Human (hg19) | 0.01 | 0.01 |
| Cow (bosTau7) | 0.49 | 0.55 |
| Goat (chir1) | 1.36 | 1.01 |

**Supplementary Figure 1: FastQ Screen analyses of PA1 at differing mismatch levels**

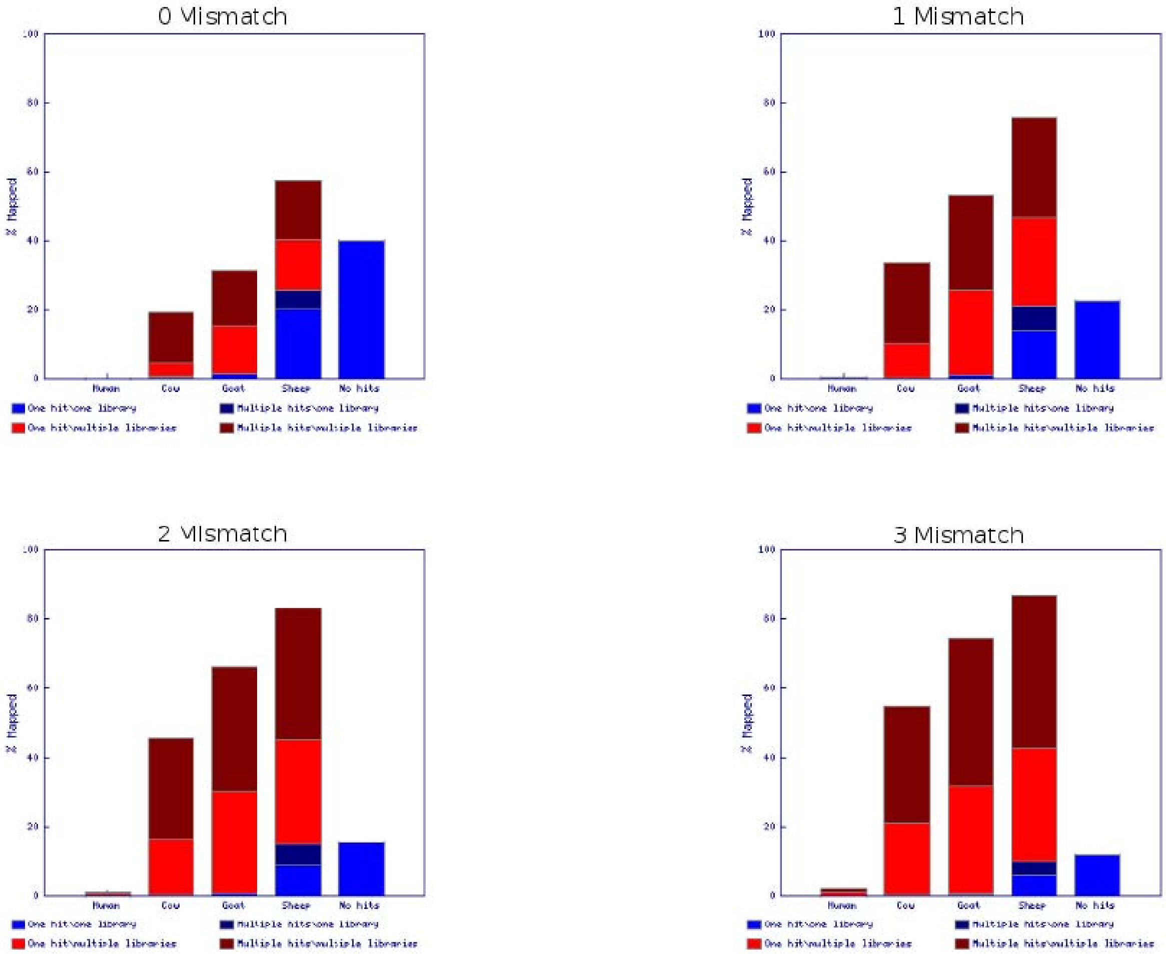

**Supplementary Figure 2: FastQ Screen analyses of PA2 at differing mismatch levels**

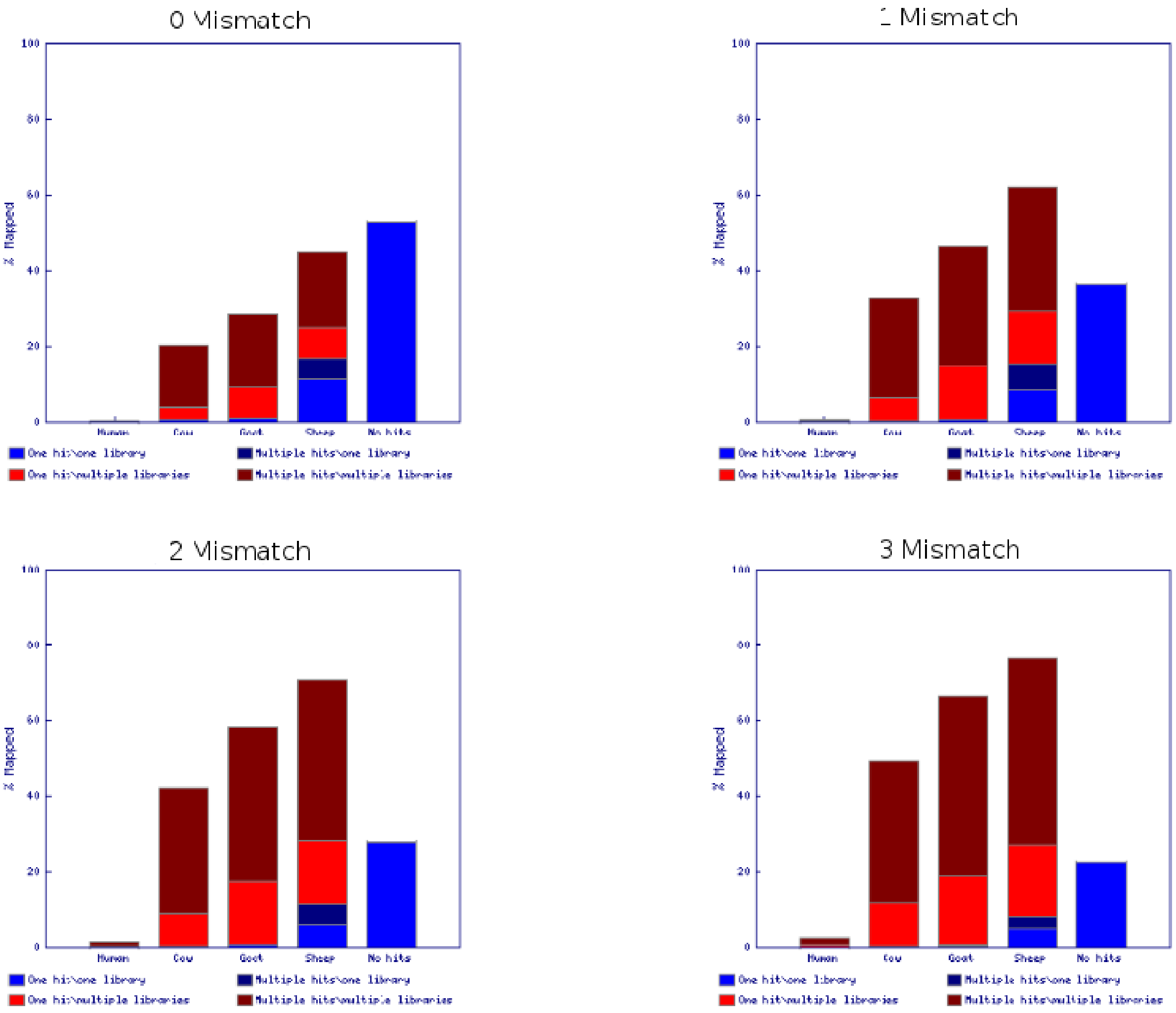

**Supplementary Figure 3: Neighbor joining tree of 23 full sheep mitochondrial sequences, showing PA1 and PA2's position inside the major haplogroup B, shown in blue**

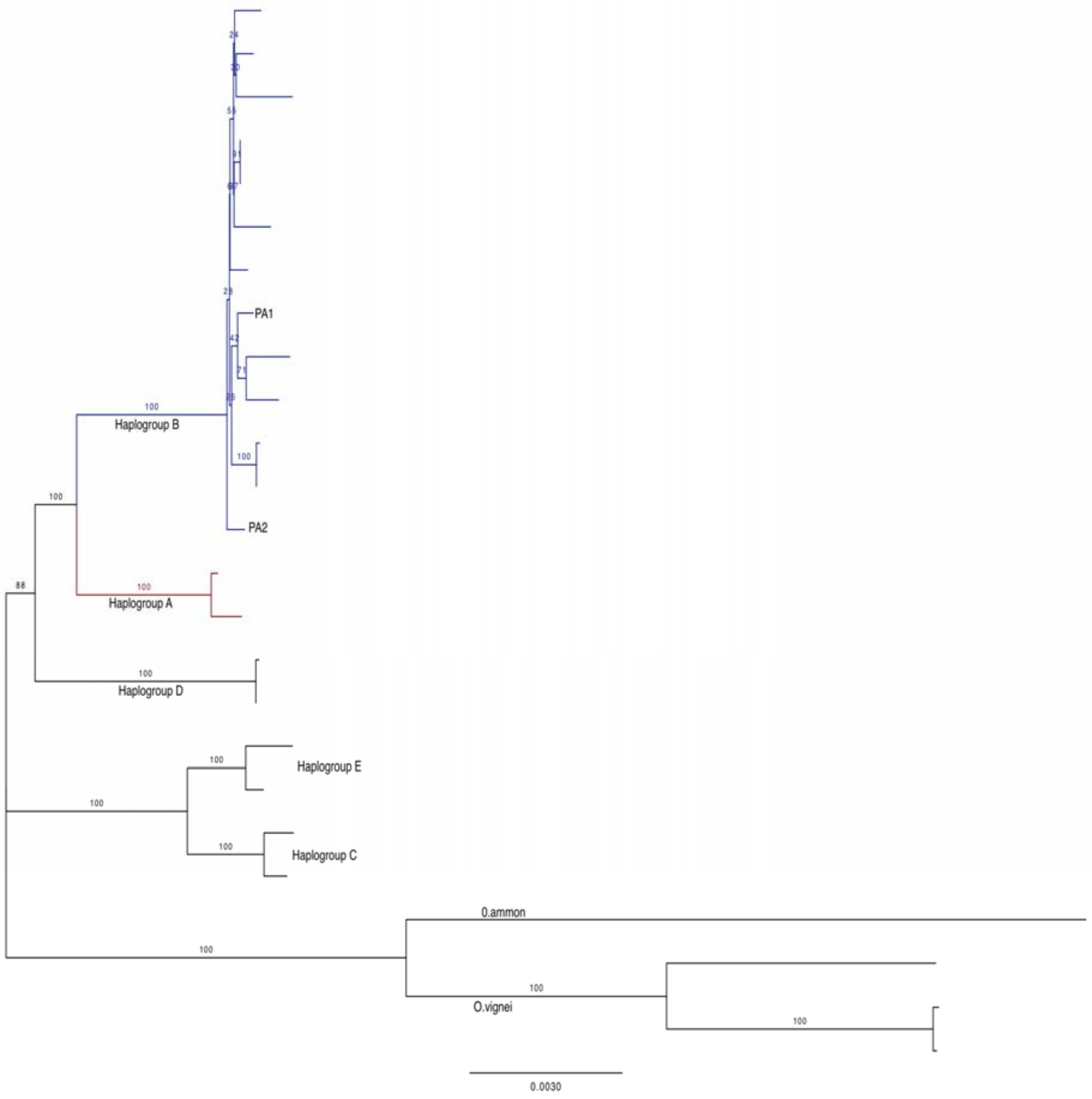

Supplement: Supplementary figures and tables [file rstb20130379supp1.pdf]
